# Common statistical concepts in the supervised Machine Learning arena

**DOI:** 10.3389/fonc.2023.1130229

**Published:** 2023-02-14

**Authors:** Hooman H. Rashidi, Samer Albahra, Scott Robertson, Nam K. Tran, Bo Hu

**Affiliations:** ^1^ Pathology and Laboratory Medicine Institute (PLMI), Cleveland Clinic, Cleveland, OH, United States; ^2^ PLMI’s Center for Artificial Intelligence & Data Science, Cleveland Clinic, Cleveland, OH, United States; ^3^ Pathology and Laboratory Medicine, University of California Davis, Sacramento, CA, United States; ^4^ Department of Quantitative Health Sciences, Cleveland Clinic, Cleveland, OH, United States

**Keywords:** Machine Learning, statistics, regression, classification, model evaluations, artificial intelligence

## Abstract

One of the core elements of Machine Learning (ML) is statistics and its embedded foundational rules and without its appropriate integration, ML as we know would not exist. Various aspects of ML platforms are based on statistical rules and most notably the end results of the ML model performance cannot be objectively assessed without appropriate statistical measurements. The scope of statistics within the ML realm is rather broad and cannot be adequately covered in a single review article. Therefore, here we will mainly focus on the common statistical concepts that pertain to supervised ML (i.e. classification and regression) along with their interdependencies and certain limitations.

## 1 Introduction

Machine Learning (ML) is now starting to make a significant impact within the healthcare domain in light of rapid developments in computational technologies and the unprecedented growth of data within this space (1–5). This massive amount of data requires enormous storage capacity and, more importantly, sophisticated methods to extract valuable information, for which the ML algorithms play a key role in.

**Figure 1:**
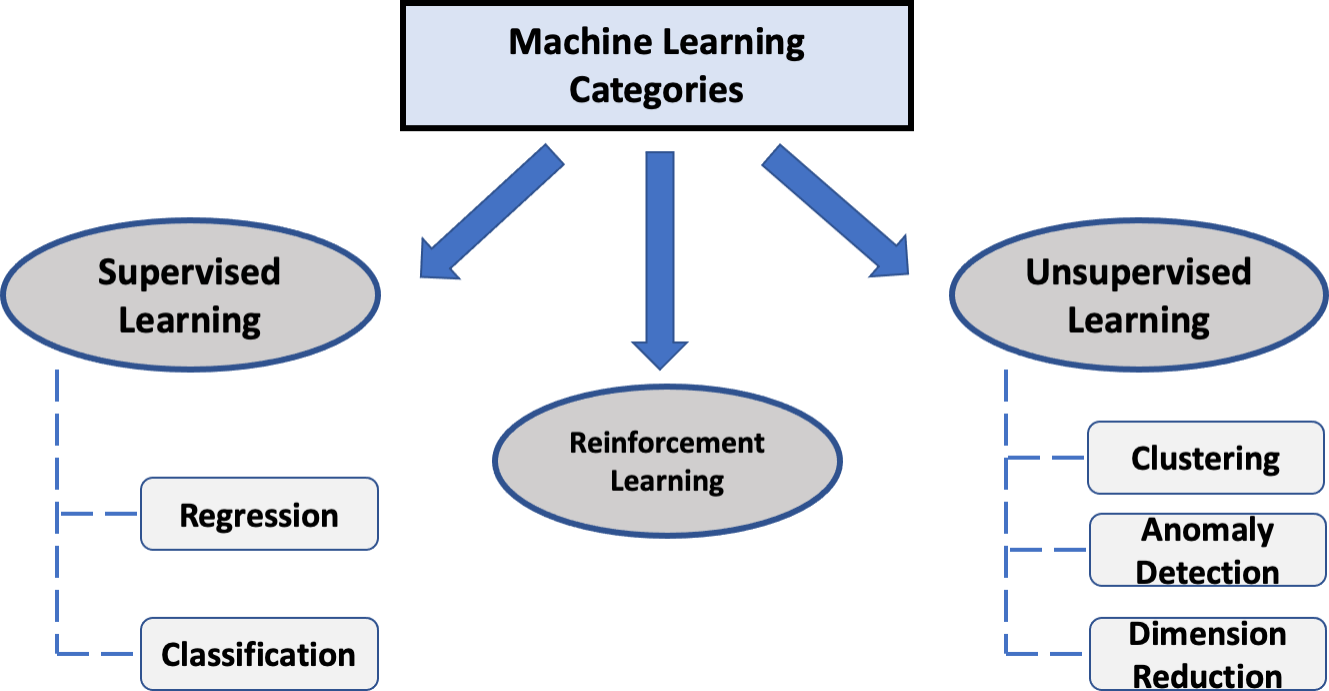
Common categories of ML algorithms.

ML is under the umbrella of artificial intelligence and its foundation is based on the disciplines of statistics and computer science, enabling it to identify inferences and relationships from data through its computationally enhanced algorithms. ML algorithms can be divided into three major categories: (i) supervised learning; (ii) unsupervised learning; and (iii) reinforcement learning (**Figure 1**).

For supervised learning, the data contains “labeled” output (or target) variable(s). A supervised ML model is then derived with the goal of predicting the output using the remaining variables (i.e., features) within the dataset by uncovering or approximating the relationship between them. Common supervised ML applications in healthcare include disease diagnosis (e.g. cancer), predicting treatment responses and certain patient outcome measures.

In contrast, the unsupervised learning approach is based on unlabeled data (i.e. no labeled output). An unsupervised learning method aims to identify subgroups or clusters of the data with same or similar patterns with little to no human intervention. It is also sometimes referred to as clustering analysis. Some of the most common unsupervised learning methods include *k*-means clustering, hierarchical clustering, principal component analysis (PCA) and anomaly detection. For more details on the unsupervised methods such as PCA, we refer to other excellent resources (6). The entities to be clustered could be either the features (columns) or the subjects (rows) of the data. One compelling application of unsupervised learning in healthcare is to redefine diseases or disease subtypes by clustering patients, which is related to certain precision medicine initiatives.

Lastly, reinforcement learning (RL) is a unique approach that incorporates a sequential decision-making process which may share certain features of both supervised and unsupervised learning. It teaches the machine *via* trial and error to learn from past experiences and adapt its actions in response to the environment, which ultimately yields the greatest reward. Although reinforcement learning at this time is not routinely used within the healthcare space, it will eventually become a major part of certain tasks as the healthcare landscape becomes increasingly more receptive to this approach. An emerging research focus of RL in healthcare is dynamic treatment regimens, which aim to identify optimal sequence of interventions for treating patients based on their characteristics and medical histories (7).

In this article we will review the common statistical concepts within the supervised ML (i.e. regression and classification) realm along with their interplay and associated limitations. Hence, we will start with the common supervised ML algorithms (regression and classification) and their related statistics followed by study design elements and practical considerations within healthcare applications.

## 2 Supervised Machine Learning: General overview

The most common supervised ML models include regression and classification (**Figure 1**). Regression deals with a continuous numerical output (e.g., blood pressure or life expectancy) while classification is for categorical outputs (e.g., discrete classes such as cancer versus normal tissue as seen in several oncology ML studies which made use of histologic images of colon, prostate and breast cancer cases). Note that the term of regression in ML varies slightly from its use in statistics, where statistical regression may also refer to some models for categorical output such as logistic regression.

The primary goal of supervised learning is to model a function underlying the statistical relationship between a set of feature variables (i.e. independent variables) and the output (i.e. target or dependent variables). For simplicity, the supervised model can be formulated as


Y=f(X)+e

where Y denotes the output, *X* = (*x*_1_,⋯,*x_p_*) denotes a *p*-dimensional vector of features, *f* represents the mathematical function that maps *X* to Y, and e represents the random error (also known as irreducible error) that is independent of *X*. Note that the true function *f* is typically unknown in practice, and can be only estimated or approximated. The above formula of supervised learning applies to most data formats (e.g., numerical tabular data, text and images). In applications with images or text, certain feature extraction methods are typically necessary to process these non-numerical raw input data into numerical tabular format. Regardless of the data, in short, the features (i.e. *X*) are mapped to the target (i.e. Y) through some acquired mathematical function (i.e. *f*).

The core step of supervised learning is to estimate or learn the function *f* based on independent samples of paired features and outputs. For instance, we can denote such a data as (*X_i_*, *Y_i_*), *i*=1,⋯, n, with *n* being the sample size, and *X_i_* and *Y_i_* being the feature and output for the *i*th sample, respectively. In general, the optimal function of *f* should be the one that minimizes the differences between the observed outputs (*y_obs_* =*Y_i_*) and the predicted outputs (*y_pred_* = *f*(*X_i_*)) across all samples. Such a difference is formally defined by a loss function in the ML literature. For regression models, the quadratic loss function is the most common choice, which is defined as $\sum {\;}_{i=1}^{n}{\left(y_{obs}-y_{pred}\right)}^{2}=\sum {\;}_{i=1}^{n}{\left(Y_{i}-f(X_{i})\right)}^{2}$ . The quadratic loss is essentially proportional to the mean squared error, MSE= $\frac{1}{n}\sum {\;}_{i=1}^{n}{\left(Y_{i}-f(X_{i})\right)}^{2}$ , which is the average difference between the observed and predicted outputs. MSE is easy to interpret and compute, and when optimized it provides a good balance between bias and variance. For classification models, a common loss function is the cross-entropy loss $-\sum {\;}_{i=1}^{n}[y_{obs\;}\log y_{pred}+(1-y_{obs})\log(1-y_{pred})]$ , which is also known as the negative log likelihood. The cross-entropy loss is typically used in conjunction with the softmax function, a common activation function for classification models that is often used in certain deep learning image classification tasks.

Once a loss function is chosen, it should be minimized with respect to the function *f*, and the optimal solution is referred to as the estimated or learned ML model. For clarification, we denote the estimated model as $\hat{f}$ in this paper to distinguish it from the true function of *f*. Additionally, to deploy the model for future prediction, it is necessary to evaluate its performance using appropriate statistical measures. Various performance metrics have been proposed for ML models including the coefficient of determination (R^2^) for regression and the area under the curve (AUC) when analyzing a receiver operating characteristics (ROC) curve for classification models (also sometimes referred to as the C or Concordance statistics). Overall, the choice of the performance metrics to use for evaluating a regression or classification model depends on the specific problem and the goals of the model. It is important to understand the limitations of each of the performance measures since no single measure should be reviewed in a vacuum. Ultimately, the choice of the performance measure should also align with the goals of the study so that it can provide the most useful information about the model’s true capabilities.

A major difference between the statistical performance measures in classification model versus a regression model is the type of output each produces. As noted, a classification model is used to predict a categorical outcome such as a yes or no response, while a regression model is used to predict a continuous numerical outcome. As a result, the types of statistical metrics used to evaluate the performance of each model are different. A classification model would use a confusion matrix-based approach to calculate metrics such as accuracy, precision, recall, and F1-score, while a regression model would use mean absolute error, mean squared error, and R^2^ (further discussed below).

### 2.1 Statistics of regression models

#### 2.1.1 Common regression algorithms

**Figure 2:**
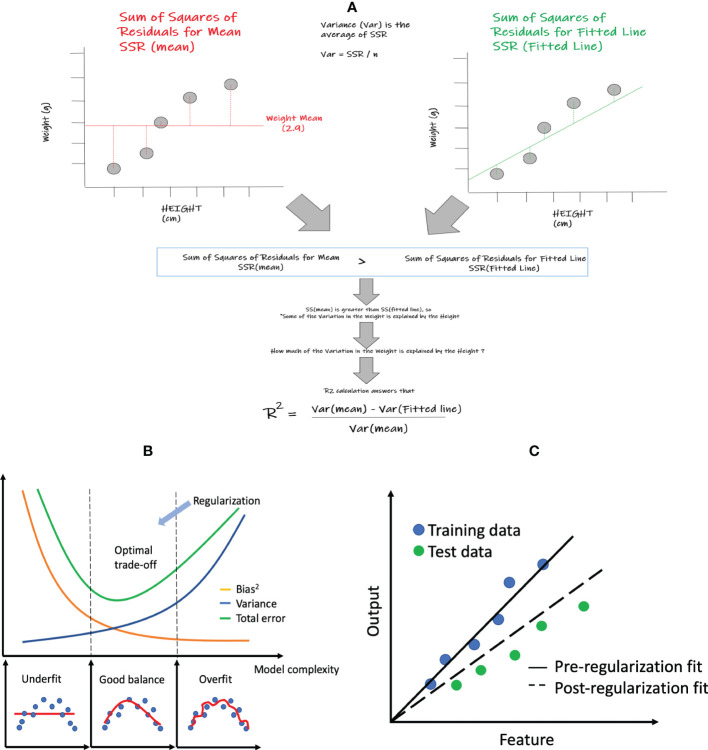
Linear regression and related statistics: **(A)** simple linear regression and the calculation of R^2^; **(B)** bias and variance tradeoff; **(C)** regularization concept: pre- and post- regularization of fitted lines.

The simplest regression model is simple linear regression, which only includes a single feature and aims to find a linear function of the feature that best predicts the dependent variable. **Figure 2A** shows an example of simple linear regression that relates height (feature) to weight (dependent variable). Although simple linear regression is not commonly employed within ML, the statistical performance measures within regression (i.e. R^2^, MSE, etc.) that are shared across many of the specific regression algorithms are easiest to comprehend within this method. Core elements within this method also enable us to better visualize and explain the relationships between the output and feature variables that are being assessed. A basic example looking at the relationship between weight and height could be a great start to better understand these core concepts and its associated model performance measures such as R^2^. In this very simple example, we will show the degree to which weight variation can be explained by height. In other words, can a best fitted line that correlates weight and height better represent this relationship than a line representing the mean weight? If the summative difference between the individual values of weight and height from the best fitted line is lower than the collective difference of the individual values of weight from the weight mean, then it could be deduced that the best fitted line can use the height values to explain some of the variations in weight better than the weight mean alone. The way we can show this mathematically is by comparing the “sum of squares of residuals” (SSR) of the mean versus the “sum of squares of residuals” of the fitted line. In the above case, the SSR of mean will be greater than the SSR of the fitted line which shows that some of the variation in the weights can be explained by heights. The next question is how much of the weight variation is explained by height, which is answered by the concept of R^2^ (See **Figure 2A**) that is further described below in section 2.1.2 below.

A deeper dive into the linear regression algorithm along with some more advanced methods such as local polynomial regression, k-NN regression, support vector regression or neural network-based regression will give us a better sense of their inner workings along with their limitations.

***Linear Regression***. The linear regression assumes a parametric linear function for the model, that is,


$$f\left(X\right)=\beta_{0}+\beta_{1}x_{1}+\cdots +\beta_{p}x_{p}$$

where *β*_0_ is the intercept and *β_j_* represents the slope associated with feature *x_j_*, *j* = 1,⋯,*p*. Estimating the function *f* thus reduces to estimating the unknown parameters *β* = (*β*_0_,⋯,*β_p_*) . In the case of a single feature (i.e., *p*=1), this reduces to the simple linear regression described above. The most common method to estimate *β* is to minimize the sum of squared residuals (SSR) or the squared loss as


$$\text{SSR}\left(\beta \right)=\sum_{i=1}^{n}{\left(y_{i}-f\left(X_{i}\right)\right)}^{2}=\sum_{i=1}^{n}{\left(y_{i}-X_{i}^{T}\beta \right)}^{2}.$$

The minimization problem above has an explicit solution of $\hat{\beta }={\left(X^{T}X\right)}^{-1}X^{T}Y$ , where ***X*** denotes the feature matrix of all subjects and ***X*** *^T^* is its transpose. $\hat{\beta }$ is called the least-square (LS) estimate in the literature. When the random error *e* follows a Gaussian distribution N(0, σ^2^), the least-square estimate is also equivalent to the maximum likelihood estimate, a more general principle in regression.

*k* ***-Nearest Neighbors.*** The *k*NN is a popular nonparametric algorithm, which uses the data points closest to the query point to make a prediction. *k*NN is a lazy learning algorithm as it does not build a complex model prior to making a prediction. Instead, it uses the training data and stores it in memory. When making a prediction for a new query point with feature *z*, it searches through the training data for the *k* nearest neighbors around *z*, computes their average, and uses the average as the prediction. *k* should be a positive integer. The closedness needs to be measured in the feature space excluding the output. The most common distance measures used are Euclidean Distance, Manhattan Distance and Minkowski Distance. For two data points with features *x* = (*x*_1_,⋯,*x_p_*) and *z* = (*z*_1_,⋯,*z_p_*), the Minkowski Distance is defined as ${\left(\sum {\;}_{j=1}^{p}|x_{j}-z_{j}{|}^{q}\right)}^{\frac{1}{q}}$ , where *q* is the order parameter that can be any real number in theory. In particular, *q*=1 is the Manhattan distance and *q*=2 is the Euclidean distance. Once the *k* closest neighbors are found for *z*, its prediction is derived as the mean outputs of these neighbors, that is, $f\left(z\right)=\frac{1}{k}\sum {i\in }_{Nz}y_{i}$ , where *N_z_* denotes the set of the *k* closest neighbors and *y_i_* denotes the outputs of the *i*th subject in *N_z_*.

The number of nearest neighbors (*k*) can be selected based on certain cross validation approaches. The *k*NN algorithm is appealing in practice for its simplicity. However, the fitted line is often jagged, especially when *k* is small (**Supplementary Figure 1**).

***Local Polynomial Regression.*** Local polynomial regression makes no global assumption about the function *f*, but assumes it as a polynomial function in moving local neighborhoods. The idea is based on the Taylor’s series expansion. For example, when the feature is univariate (i.e., *p*=1), the local polynomial regression with an order *m* can be expressed as


$$f\left(X\right)=\sum_{j=0}^{m}\alpha_{j}{\left(X-z\right)}^{j}$$

for any data *X* close to *z*, the query point of interest; *α*_0_ = *f*(*z*) and *α_j_*’s are the unknown coefficients for the other polynomial terms. The local polynomial regression can be estimated by generalizing the least-square estimate for linear regression to minimizing the kernel-weighted local sum of squared residuals as


$$\text{SSR}\left(f,\;z\right)=\sum_{i=1}^{n}K\left(X_{i},\;z\right){\left(y_{i}-f\left(X_{i}\right)\right)}^{2},$$

where *K*(·,·) is a kernel function that assigns weights to the training data points. The data points closer to *z* should get greater weights than those further away from it. A commonly used kernel is the Gaussian kernel, which has the function of


$$K\left(X,\;z\right)=\frac{1}{\sqrt{2\pi }\sigma }\exp\left(-\frac{||X-z|{|}^{2}}{2\sigma^{2}}\right).$$

If we assume that the function *f* is a constant (i.e., *m*=0) in the neighborhood, the resulting estimate becomes the Nadaraya-Watson estimate. The *k*NN algorithm can be viewed as a special Nadaraya-Watson estimate, where the kernel function equals 1 for the *k* nearest neighbors and equals 0 for the other data points. **Supplementary Figure 1** shows the fitted lines of the Nadaraya-Watson estimate and the local polynomial regression with a degree of 3 (i.e., local cubic regression). The local polynomial regression method is useful when the underlying data is nonlinear and there is no applicable parametric model. It can also be used to identify local patterns in the data which may not be evident in a global model.

***Support Vector Regression***. Support vector regression (SVR) is an extension of the Support Vector Machine (SVM) algorithm (used for classification problems) that enables regression tasks. More details about SVM are described in section 2.2.1. SVR uses similar principles as SVM, but is used for continuous outputs.

***Neural Network Regression*.** Neural network regression is based on a network of artificial neurons that may use a variety of techniques like back-propagation, dropout and adaptive learning to adjust weights and thresholds in order to achieve the desired accuracy. The weights and thresholds are adjusted using formulas such as the Gradient Descent, the Adam Optimizer, and the root mean squared error. These formulas are used to calculate the error between the predicted and actual values and to update the weights and thresholds accordingly. Neural network regression is an effective ML technique that can be used to predict continuous values with great accuracy.

#### 2.1.2 Performance metrics for regression

The most common statistical measures for evaluating the performance of regression are Mean Absolute Error (MAE), Mean Squared Error (MSE), Root Mean Squared Error (RMSE), R^2^, and adjusted R^2^.

The Mean Absolute Error (MAE) (related to the L1 loss) is the average absolute difference between the predicted and observed (true) outputs. It is calculated as $MAE=\frac{1}{n}\sum {\;}_{i=1}^{n}|y_{i}-{\hat{y}}_{i}|$ , where *y_i_* and ${\hat{y}}_{i}=\hat{f}\left(X_{i}\right)$ represent the observed (true) and predicted outputs for the *i*th sample, respectively.

The Mean Squared Error (MSE) (also known as the L2 loss) is calculated as the sum of the squared differences between the predicted and observed outputs, divided by the number of samples, $MSE=\frac{1}{n}\sum {\;}_{i=1}^{n}{\left(y_{i}-{\hat{y}}_{i}\right)}^{2}$ . MSE is more sensitive to outliers than MAE, as it penalizes large errors more heavily.

Root Mean Squared Error (RMSE) also known as the root mean squared deviation is the square root of the MSE. It is commonly used as it has the same units as the original data, making it easier to interpret. MSE or RMSE can be used to compare the performance of different regression models, with a lower value indicating a better fit. However, it is important to note that they are sensitive to the scale of the target variable. Thus, they are generally not applicable for comparing the models with different target variables.

*R* ^2^ is the coefficient of determination and defined as


$$R^{2}=1-\frac{\text{SSR}}{SSmean}=1-\;\frac{\sum_{\text{i}=1}^{\text{n}}{\left(y_{i}-\;{\hat{y}}_{i}\right)}^{2}}{\sum_{\text{i}=1}^{\text{n}}{\left(y_{i}-\bar{y}\right)}^{2}}$$

where $\bar{y}=\frac{1}{n}\sum {\;}_{i=1}^{n}y_{i}$ is mean of all observed outputs and $\text{SSmean}=\sum {\;}_{\text{i}=1}^{\text{n}}{\left(y_{i}-\bar{y}\right)}^{2}$ is referred to as the sum of squares of the mean (**Figure 2A**), which is equivalent to the sum of squared residuals from the sample mean. Since SSmean is always greater than or equal to SSR, *R* ^2^ has a value between 0 and 1 and can be interpreted as the proportion of the variance of the target variable that is explained by the features in the model. In the case of a single feature, *R* ^2^ is the square of the Pearson correlation coefficient between the output and the feature. A *R* ^2^ of 0 indicates that the model does not explain any of the variance in the output variable and a value of 1 indicates that the model explains all of the variance. Overall, *R* ^2^ is useful for assessing the overall fit of a regression model, while MAE, MSE, and RMSE are more useful for assessing the performance of a model on a particular dataset. One limitation of *R* ^2^ is that it will increase when extra features are added into the model. Therefore, it may be not useful to compare models with nested sets of features.

Adjusted *R* ^2^ is a modification to account for this issue, which is defined as


$$R_{adj}^{2}=1-\frac{\frac{\text{SSR}}{n-p-1}}{\frac{SSmean}{n-1}}$$

where *n*-*p*-1 and *n*-1 are the degrees of freedom for SSR and SSmean, respectively. The adjusted R^2^ basically penalizes the models for adding features not related with the output variable, which is thus less biased than R^2^. The value of $R_{adj}^{2}$ is always less than or equal to *R* ^2^.

#### 2.1.3 Bias and variance trade-off and regularization

The bias-variance tradeoff is a central concept in supervised ML studies. It states that an algorithm’s ability to generalize to unseen data is a tradeoff between its complexity (variance) and its bias. Bias refers to the error that is introduced by simplifying the model, while variance refers to the error that is introduced by making the model too complex. When an algorithm has low bias, it is generally more complex (i.e. increased variance) and more likely to overfit the training data. Conversely, when an algorithm has high bias (i.e. low variance), it may be too simple and is more likely to underfit the training data (8). Mathematically, for the query data point with feature *z*, the true and predicted outputs are *f*(*z*) and $\hat{f}\left(z\right)$ , respectively, and the total expected error can be expressed as

$E{\left(f\left(z\right)-\hat{f}\left(z\right)\right)}^{2}=E{\left(f\left(z\right)-E\hat{f}\left(z\right)+E\hat{f}\left(z\right)-\hat{f}\left(z\right)\right)}^{2}={\left(E\hat{f}\left(z\right)-f\left(z\right)\right)}^{2}+Var\left(\hat{f}\left(z\right)\right)=bias^{2}+variance,$

where the first term represents the squared bias, and the second term represents the variance of the prediction itself. As shown in **Figure 2B**, as the model becomes more complex, the bias is reduced but the variance increases. Thus the model deviates from the optimal spot with the lowest total prediction error.

One popular method to strike a balance between the bias and variance is regularization. Regularization addresses the bias-variance tradeoff by adding a penalty to the loss function of a model to reduce its variance at a sacrifice of a small amount of bias. Mathematically, regularization for regression can be formulated as minimizing the penalized sum of squared residuals,


$$\sum_{i=1}^{n}{\left(y_{i}-f\left(x_{i}\right)\right)}^{2}+\lambda R\left(\beta \right),$$

where *λ* is the tuning parameter and *R*(*β*) is a penalty term. There are three common penalty terms in practice: L2 norm of the parameters with $R\left(\beta \right)=\sum {\;}_{j=1}^{p}\beta_{j}^{2}$ for ridge regression, L1 norm with $R(\beta )=\sum {\;}_{j=1}^{p}\left|\beta_{j}\right|$ for least absolute shrinkage and selection operator (LASSO) (9), and a combination of L1 and L2 norms as $\lambda_{1}\sum {\;}_{j=1}^{p}|\beta_{j}|+\lambda_{2}\sum {\;}_{j=1}^{p}\beta_{j}^{2}$ for elastic net (10). The lambda parameters can be selected with cross validation. When LASSO or elastic net is used, some regression coefficients may be shrunken to zero so that they can be also considered as feature selection procedures.

Regularization can help improve the generalization of a model, making it more resilient to overfitting. **Figure 2C** shows that the model without regularization fits the training data very well but has large prediction errors when applied to the testing data. After regularization, the model achieves more balanced performance though the bias is larger for the training data.

Other methods for optimizing the bias-variance tradeoff in supervised learning tasks include the use of ensemble techniques, early stopping, and feature selection. Additionally, hyperparameter tuning with certain cross-validation tasks can also reduce overfitting and improve the generalization performance of an ML model.

### 2.2 Statistics of classification

#### 2.2.1 Common classification algorithms

**Figure 3:**
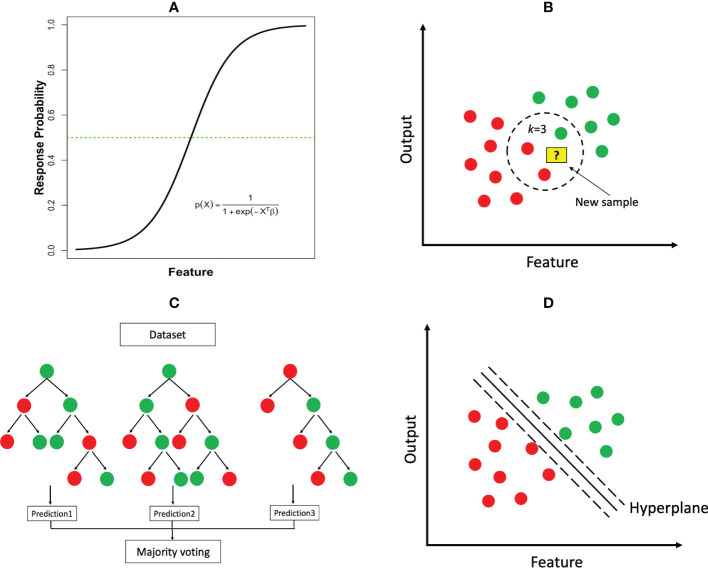
Common ML classification algorithms: **(A)** logistic regression; **(B)** *k*-nearest neighbor (*k*=3); **(C)** random forest; **(D)** support vector machine.

***Logistic Regression.*** Logistic regression is not typically used in image or text classification tasks but arguably one of the most popular methods for classification within the tabular data domain. It is used to predict the probability of the output (e.g., the probability that a patient has a cancer based on various features such as age, blood pressure, and genomic mutations). The model estimates the probability using the logistic function, which takes the following form:


$$P\left(X\right)=\frac{\exp\left(\beta_{0}+\beta_{1}X_{1}+\cdots +\beta_{p}X_{p}\right)}{1+\exp\left(\beta_{0}+\beta_{1}X_{1}+\cdots +\beta_{p}X_{p}\right)}=\frac{1}{1+\text{exp}\left(-X^{T}\beta \right)}$$

where *P*(*X*) represents the probability of the outcome occurring (e.g. cancer diagnosis); *X* = (*X*_1_,⋯,*X_p_*) and *β* = (*β*_1_,⋯,*β_p_*) represent the features and associated parameters as in linear regression. **Figure 3A** illustrates the logistic function in the case of a single feature. An equivalent transformation of the logistic model is to express it in terms of log-transformed odds as $\text{log}\frac{P\left(X\right)}{1-P\left(X\right)}=\beta_{0}+\beta_{1}X_{1}+\cdots +\beta_{p}X_{p}$ , where each *β* parameter can be interpreted as log-odds ratio of the associated feature, a measurement widely used in the medicine literature. In the case that the output is a multi-class (i.e., more than two classes) variable, the logistic model has two common extensions, multinomial logistic regression and ordinal logistic regression, where the latter is tailored to the output variable whose classes have a natural order (e.g., mild, moderate and severe conditions).

*k* ***-Nearest-Neighbors.*** The *k*NN algorithm can be used for both regression and classification tasks. This is a type of instance-based learning where the model is trained by storing the grouped training data and making predictions based on the *k* nearest neighbors. To make a prediction for a new sample, the algorithm calculates the distance between the new sample and each of the stored training samples according to their features, and determines the *k* closest samples. The prediction is then based on the majority class of the *k* nearest neighbors (**Figure 3B**).

***Naïve Bayes.*** Naive Bayes classifiers are a collection of classification algorithms that use the Bayes theorem. Following the Bayes theorem, the conditional probability of the output *Y* given the feature *X* = (*X*_1_,⋯,*X_p_*) can be written as $p\left(Y|X\right)=\frac{p\left(Y,X\right)}{p\left(X\right)}=\frac{p\left(X|Y\right)p\left(Y\right)}{p\left(X\right)}.$ The naïve Bayes methods namely make a naïve assumption that the features are independent of each other. Under this assumption, the above expression can be further decomposed as


$$p\left(Y|X\right)=\frac{p\left(X|Y\right)p\left(Y\right)}{p\left(X\right)}=\frac{p\left(X_{1}|Y\right)\ldots p\left(X_{p}|Y\right)p\left(Y\right)}{p\left(X_{1}\right)\ldots p\left(X_{P}\right)}.$$

Different naïve Bayes classifiers differ mainly at the distribution of the features given the output (i.e., *p*(*X_j_* |*Y*), *j* = 1,⋯,*p*). Depending on the distributions applied, common classifiers include Gaussian naïve Bayes, multinomial naïve Bayes and Bernoulli naïve Bayes classifiers. Despite the incorrect assumption of features being independent of each other, naive Bayes classifiers can sometimes produce reasonable results, especially for simple tasks. However, their performance has been shown to be inferior to some of the other well-established algorithms for more complex tasks.

***Decision Tree Based Methods.*** These methods can be used for both classification and regression tasks. A decision tree is a model that partitions the feature space into distinct regions hierarchically. A classification decision tree starts with a root node, which is then divided into a left child and a right child. Each child node is further split to create successive partition. A child node that cannot be subdivided is ultimately called a leaf node (**Figure 3C**), where the final class label is assigned. The construction of a tree requires determining the feature to split and the split cut-off value at each node, a termination rule and how to label each terminal node. Impurity measures such as cross-entropy and Gini index are common criteria to determine these elements. Once a tree is constructed, predictions can be made by following the set of split rules from the root to the leaves. The size of the tree (i.e., the number of nodes) represents the complexity of the model. A very large tree may overfit the data, for which tree pruning will be needed.

A single classification tree is rarely used in practice since it can be highly variable. The more advanced tree-based methods may overcome these limitations; these include ensemble methods such as the bootstrap aggregating (bagging) approach, random forest (RF) and gradient boosting machine (GBM). Bagging constructs a large number of trees with bootstrapped samples from the data, and a classification will be made by aggregating over the results from individual trees based on a majority vote (11). *Random forest* is very similar to bagging (12). Namely, RF grows trees by introducing randomness to the modeling process. Before each node is split, RF randomly selects a subset of features as candidates instead of searching all features. This introduces a wider diversity and the trees in RF become more independent than those in bagging. Boosting is another powerful ensemble method (13). Unlike bagging and RF that construct a larger number of trees in parallel, boosting (as seen in GBM) creates multiple trees sequentially. Each tree is grown based on the information from previously grown trees (i.e., weak learners) in order to reduce the errors from the previous trees, which can ultimately lead to a better performing model (i.e., a boosted tree). Boosting sometimes yield very reasonable models, especially for unbalanced data sets. However, their limited number of tuning parameters may sometimes make boosting more prone to overfitting compared to RF that uses a larger number of parameters for tuning and model optimization (1).

***Support Vector Machine (SVM).*** The central idea of support vector machines is to identify a hyperplane as a boundary to separate the output classes as wide as possible. When there are only two features, the separating hyperplane reduces to a linear line (**Figure 3D**). The separation is maximized by increasing the margin on either side of the line. However, in many cases, it is impossible to find such a linear hyperplane that perfectly separates the two output classes in the original feature space. Fortunately, a powerful highlight of SVM is to project the data into a higher dimensional space using a nonlinear *kerne*l (i.e. a mathematical “function”) and then search a separating hyperplane in the newly projected space. The resulting hyperplane will be nonlinear when transformed back to the original feature space. This classic use case of Kernels (e.g. the RBF kernel) as seen in SVM, ultimately enhances the algorithm’s capability of finding a linear decision boundary for tasks that would have otherwise not been separable in lower dimensions. The overall process may also help to improve the final model’s performance in several ways which include, separability, dimensionality reduction, regularization, and computational efficiency.

Common choices of the kernel function in SVMs include:

- Polynomial kernel with order *q*: *K*(*x*, *x* ^′^)=(1 + 〈*x*, *x* ^′^〉)^*q*^ ,
- Radial basis kernel: *K*(*x*, *x* ^′^)=*exp*(−*γ*||*x*−*x* ^′^||^2^) ,
- Neural network kernel: *K*(*x*, *x* ^′^)=*tanh*(*k*_1_||*x*−*x* ^′^||+*k*_2_) .

SVM can yield highly accurate prediction using the flexible kernel functions. By introducing a cost parameter to loosen the perfectness of separation, SVM is also relatively robust to outliers. However, training SVMs could be computationally expensive on large datasets. It can also be viewed by some as a black box approach (especially incorporating certain kernels) since the separation of classes may not be intuitive.

***Neural Network***. Neural networks are increasingly being used in both regression and classification tasks. These are inspired by the structure and interplays between human neurons and typically include an input layer (receiving the raw input data), hidden layers and an output layer (producing the final output of the model). Two major neural networks used regularly within medicine include the Multilayer perceptron (MLP) and the convolutional neural network (CNN). MLPs are typically used for tasks involving structured data (i.e. tabular numerical data), while CNNs are better suited for tasks involving unstructured data, such as images for classification or object detection tasks. These CNN deep neural network approaches are sometimes also referred to as deep learning.

#### 2.2.2 Convolutional neural networks and object detection

As noted, a CNN is a special neural network with different specific layers (i.e. input layer, convolutional layer, pooling layer, and full-connected layer). **Figure 5A** illustrates the architecture of the CNN. The convolutional layer is the core building block of the CNN, and there could be a series of convolutional layers present. When stacked on top of each other, convolutional layers can detect a hierarchy of imaging features and patterns. These features are then pooled and fed into the fully connected layers of artificial neurons for various classification tasks.

Although, these black box neural network methods may be hard to understand, their inner workings are very much based on some traditional statistical concepts. For example, in a CNN, the logit function (AKA sigmoid function) is used to map the input values to a 0 to 1 range and typically serves as the last step before the final output of a CNN. This function is used to calculate the probability of each class and to assign the class (e.g. 0 class or class 1) with the highest probability to the final output. Notably, the logit function in a CNN and the logistic regression algorithm are related in the sense that both use the logistic function (AKA sigmoid function) as noted above which enables both to acquire and assign the relationship between the input variables and the output variable.

In recent years, with the development of deep learning, CNN-based models have made great breakthroughs and have become the gold standard for most image-based tasks (14). The Image-based ML tasks can be categorized as image classification, image generation, object detection and image segmentation.

Image classification involves training a neural network to assign an input image to a specific class or category based on the whole image. For example, an image classification model might be trained to recognize various types of cancer (such as colon, breast, and prostate cancer). The model would be trained on a large dataset of labeled images from colon, breast, and prostate cancer which will then allow the CNN to learn and recognize patterns and features in the images that are characteristic to each of the assigned classes (colon, breast, and prostate). The end result is the trained model that will be able to classify new images based on their shared characteristics to the labeled target class.

Object detection is related to image classification, but its goal is to identify and locate objects within an image (rather than a global analysis or classification of the image). This involves identifying the location and bounding box of each object in the image, as well as classifying each object into a specific class. An example of an object detection model within medicine is one that can detect and identify various individual white blood cells (e.g. neutrophils, lymphocytes, monocytes, eosinophils and basophils) in the peripheral blood (15).

Performance measures for object detection and image classification have some similarities, but also have some key differences. Additionally, the type of image classification (binary versus multi-class) may also influence certain performance measures which will need to be accounted for (further discussed below).

#### 2.2.3 Performance metrics for binary classification

Regardless, if it’s an image or tabular data task, the performance of a classification model within these studies can be evaluated using numeric metrics (e.g. accuracy, etc.) along with graphical representations (e.g. the ROC curve, etc.).

**Figure 4:**
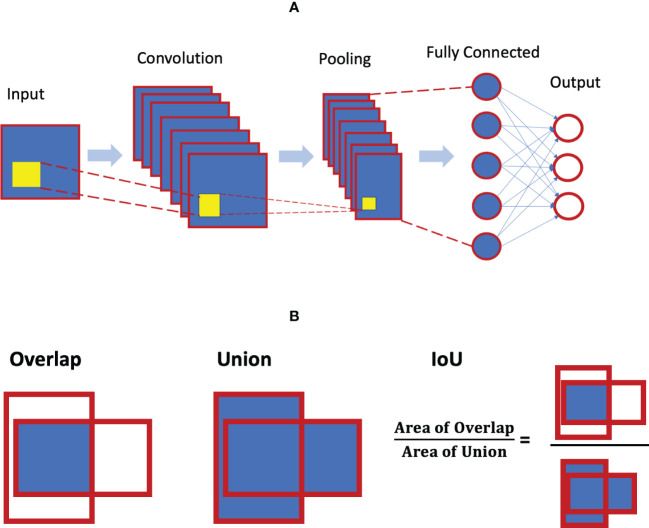
**(A)** Convolution neural network architecture concept; **(B)** concept of intersection over union (IoU) for object detection.

The performance measures of a classification ML model are derived from a confusion matrix-based approach. A confusion matrix tabulates the predicted outputs as they relate to the observed (true class) outputs, yielding the numbers of true positive, true negative, false positive, and false negative predictions made by the model (**Figure 4A**). A true positive (TP) prediction is when the model correctly predicts that a given sample belongs to a positive class while a true negative (TN) prediction is when the model correctly predicts that a given sample belongs to a negative class. However, the model will likely also make some mistakes since no ML model is perfect. The mistakes are presented as the false positive and the false negative predictions. A false positive (FP) prediction is when the model incorrectly predicts that a given sample belongs to a positive class, when it actually belongs to a negative class while a false negative (FN) prediction is when the model incorrectly predicts that a given sample belongs to a negative class, when it actually belongs to a positive class.

The numbers of TP, TN, FP and FN cases are then used to calculate several key statistical performance measures (accuracy, precision, sensitivity, specificity, F1, etc.) that display the model’s ability to distinguish between the positive and negative cases (**Table 1**). The first metric is accuracy, which is the percentage of correct predictions (i.e. (TP + TN)/(TP + TN + FP + FN)). While the meaning of accuracy is very intuitive, it has a major drawback for imbalanced datasets, especially when one output class is much more common than the other class. In this case, accuracy mainly depends on the performance for the biggest output class, and a naive model that predicts all data into the dominant class could display a high accuracy. The balanced accuracy is a better alternative for imbalanced datasets, which gives equal weights to the classes. It is defined as an average of the percentages of correct predictions in each class.

**Table 1:** Evaluation metrics for binary classification based on the confusion matrix.

Metric	Formula
Accuracy	$\frac{TP+TN}{TP+TN+FP+FN}$
Sensitivity/recall*	$\frac{TP}{TP+FN}$
Specificity*	$\frac{TN}{TN+FP}$
Precision/positive predictive value	$\frac{TP}{TP+FP}$
Negative predictive value	$\frac{TN}{TN+FN}$
Balanced accuracy	(Sensitivity + Specificity)/2
F1 score	$\frac{2}{Precision^{-1}+Recall^{-1}}=\frac{TP}{TP+0.5\left(FP+TN\right)}$
Matthews Correlation Coefficient	$\frac{TP\times TN-FP\times FN}{\sqrt{\left(TP+FN\right)\left(TP+FP\right)\left(TN+FN\right)\left(TN+FP\right)}}$
Cohen’s Kappa	$\frac{Oberserved\;accuary-expected\;accruacy}{1-expeceted\;accuracy}=\frac{2\times \left(TP\times TN-FP\times FN\right)}{\left(TP+FP\right)\left(TN+FP\right)+\left(TP+FN\right)\left(TN+FN\right)}$

*Are Independent of the prevalence rate of the output.

Other metrics that can be directly computed from the four components of the confusion matrix include sensitivity (also referred to as positive recall or true positive rate), specificity (also referred the true negative rate), positive predictive value (PPV, also referred to as precision) and negative predictive value (NPV), each measuring a particular aspect of the model’s prediction performance. Sensitivity is the proportion of correct predictions among those who are truly positive (i.e. true positive rate), while the specificity is the proportion of correct predictions among the true negatives (true negative rate). The balanced accuracy (mentioned above) is essentially the average of sensitivity and specificity for binary classification. PPV and NPV are the correct proportions among the predicted positives and negatives, respectively. The F1 score is a metric reflecting the overall performance, which is calculated as the harmonic mean of recall (sensitivity) and precision (PPV). By its definition, the F1 score varies with class swapping, and it has nothing to do with the number of correctly predicted negatives. These metrics all have values in the range between 0 and 1, with higher values representing better performances for that particular metric.

The Matthew’s correlation coefficient (MCC) and the Cohen’s kappa are two other metrics for the overall performance of an ML model. MCC is a considered a reliable and balanced statistical metric in the sense that it produces a high score only if the prediction yields good results in all of the four components of the confusion matrix (16). The Cohen’s kappa is another metric that has become increasingly popular (17). Both MCC and kappa have values between -1 and 1. Values close to 1 indicate good agreement and a value of 0 implies that the classification model is basically equivalent to complete random guess. Negative values indicate the classifier is even worse than chance, which may imply certain major errors in the model development process.

These metrics provide a summary of the model’s performance and can also be useful for comparing the performance of different models, highlighting areas where the model is performing well (e.g. high sensitivity) in certain tasks and poorly in others (e.g. low specificity). This confusion matrix approach can also be applied (with slight modification) to a multi-class approach such as in distinguishing colon versus breast versus prostate cancer cases (discussed in more detail in the multi-class section below).

It is also important to note that except for sensitivity and specificity, many of these performance metrics are prevalence dependent. Additionally, it is essential that we do not evaluate these in a vacuum since there are key interdependencies between many of these performance measures. For example, certain performance measures trend in similar directions with changing thresholds (e.g. sensitivity and negative predictive value move together in one direction while specificity and precision move together in another direction with a changing model threshold). Additionally, certain values follow opposite trends (e.g. increased sensitivity is usually at the cost of a decreased specificity and increased negative predictive value is usually at the cost of the deteriorating positive predictive value).

For classification algorithms that generate probabilities but not direct labels for the output variable (e.g., logistic regression), a cut-off threshold is needed for the final output prediction. A naïve choice would be 0.5 (which is the default choice for many ML models at baseline), that is, a case is predicted if the probability is greater than 0.5. In theory, we can choose any cut-off value between 0 and 1. A higher threshold (>0.5) will usually reduce the FP rate while a lower threshold (<0.5) will usually reduce the FN rate. In practice, the choice of the optimal cut-off value may depend on the study goals. For example, a low cut-off may be applied to a diagnostic test for a deadly disease to reduce the false negative rate. In contrast, a high cut-off may be used if the test is risky and costly.

If one wishes to evaluate the classifier without having to select a specific threshold, such an evaluation can be achieved and visualized using the ROC curve (**Figure 4B**). A ROC curve is constructed by computing the sensitivities and specificities at many possible cut-off values, and then plotting the sensitivities against one minus the specificities (i.e. the false positive rate; FPR). The area under the ROC curve (ROC-AUC) is a commonly used metric to evaluate the classification model’s global performance, which reflects the overall ability of the model to separate the output classes. The ROC-AUC score ranges from 0 to 1, with a value of 1 indicating perfect performance and a value of 0.5 indicating random or no discriminatory power. Generally, the closer to 1 you get the better. It is important to note that the interpretation of ROC-AUC scores will depend on the specific context and goals of the model. In some cases, a lower ROC-AUC score (e.g. 0.75) may still be acceptable if it meets the needs of the application. In other cases, a higher ROC-AUC score (e.g. 0.9) may be required in order to achieve acceptable performance.

Like the other performance measures, ROC-AUC should not be used as the only performance measure since it too has its shortcomings. One major limitation of using ROC curve is the impact of class imbalance. For instance, a classification model can easily get a high AUC for a rare case scenario even with a very low true positive rate. A complement is the precision-recall (PR) curve (**Figure 4C**). In contrast to the ROC-AUC in which the better performing models gravitate to the top left of the ROC curve, the goal in PR curve optimization is to get close to the top-right corner of the curve.

In addition to the confusion matrix-based performance metrics noted above, in many cases it becomes vital to also assess the accuracy of the actual underlying probability score (a non-confusion matrix measure) that was ultimately used to render the outcome. A calibration curve and Brier score can fulfil this purpose. Calibration is examined by comparing the distribution of the predicted probabilities with that of the observed or empirical probabilities. This can be achieved by plotting them in quantile buckets, yielding a calibration curve (**Figure 4D**). Miscalibration occurs if the calibration curve deviates significantly from the diagonal line. Note that not all classification models are able to generate probabilities for prediction. Another measure that can complement the calibration curve is the Brier score. The Brier score is a measure of the accuracy of probabilistic predictions. It is defined as the mean squared difference between the predicted probabilities and the actual outcomes, where 0 is a perfect score and higher values indicate worse calibration.

#### 2.2.4 Performance metrics for multi-class classification

The performance metrics for binary classification can be readily extended to multiclass classification. The confusion matrix for multi-class classification expands to a *K*-by-*K* matrix with *K* being the number of classes. **Supplementary Figure 2** shows a confusion matrix for a multiclass classification example with *K*=3 classes (specifically distinguishing colon, prostate and breast cancer). The extensions of the performance metrics based on the confusion matrix generally follow three different approaches noted below (18).

The “micro” approach computes the metrics globally. For example, the accuracy is the overall percentage of correct prediction, which is the ratio of the sum of diagonal elements of the confusion matrix over the total sample size. The “macro” approach starts by decomposing the multiclass classification into *K* independent binary classifications, each for separating one particular class from the rest classes. This is referred to as “one versus rest” (OvR) approach in the literature. Correspondingly, the *K*-by-*K* confusion matrix can be transformed into *K* 2-by-2 confusion matrices, one for each binary classification (**Supplementary Figure 2**). The macro metric is basically the mean of the metrics derived from these *K* matrices. For example, the sensitivities are 0.56, 0.5 and 0.73 for classifying colon, prostate and breast, respectively. The macro sensitivity is then (0.56 + 0.5+0.73)/3 = 0.6. The “weighted” approach is similar to the macro approach, but the mean over all classes is weighted by the frequency (or prevalence rate) of each class. Thus, the weighted sensitivity for this example is 0.59 (see **Supplementary Figure 2**).

The F1 score for multiclass classification has the same formula as for binary classification and relies on the recall and precision being used. For instance, the macro F1 score is the harmonic mean of the macro average recall and the macro average precision. The Matthew’s correlation coefficient (MCC) and the Cohen’s kappa can be computed directly from the *K*-by-*K* confusion matrix for multiclass classification. MCC and Cohen’s kappa share the same numerator (**Supplementary Table 1**), but kappa has a smaller denominator and is usually greater than MCC.

ROC and PR curves can be constructed for multiclass classification using two approaches. The first approach is to follow the OvR principle to create multiple curves for *K* binary classification, each for classifying one class against the rest. The area under the curve is then the average of the areas of the individual ROC or PR curves. The other approach is referred to as “one versus one”, which creates corresponding curves for all pairwise combinations of classes. The areas under these curves are then averaged.

In contrast to the aforementioned general classification tasks, certain ML approaches (e.g. object detection) may require their own unique set of performance measures.

#### 2.2.5 Performance metrics for object detection

In object detection, the goal is to accurately identify and locate the object of interest within an image. This might involve identifying the location and bounding box of each object in the image, as well as classifying each object into a specific category. In general, evaluating the overall classification in object detection can employ the same metrics for binary or multi-class classification, however in many cases certain confusion matrix elements (e.g. true negative cases) may not be readily available due to the intrinsic nature of this approach. Regarding object localization, a common measure is the average precision (AP: calculated as the area under the PR curve) at different intersection over union (IoU) thresholds (described below). The mean average precision (mAP: calculated by averaging the AP over all objects and/or thresholds) is then used to quantify the model’s accuracy at identifying and locating the objects within an image.

**Figure 5:**
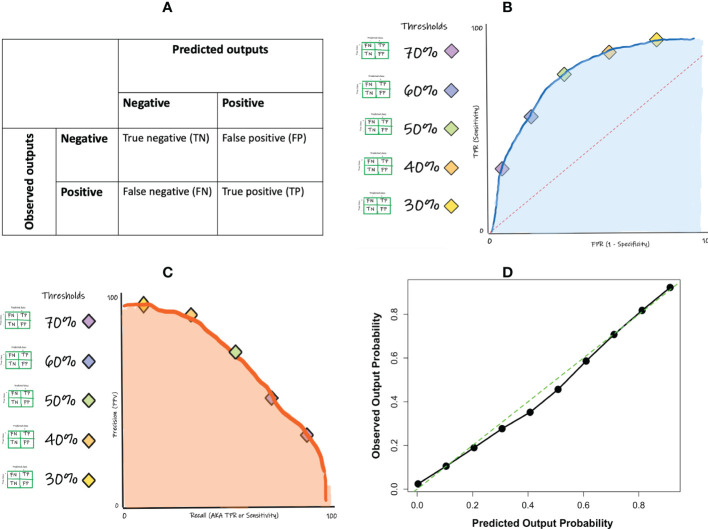
Performance metrics for binary classification: **(A)** confusion matrix concept; **(B)** ROC curve concept **(C)** precision-recall curve concept; **(D)** calibration curve example.

The metric of intersection over union (IoU) is considered to be the gold standard for evaluating object localization in the literature. The localization of an object is typically quantified as a bounding box that provides the coordinates of that object. The shape of the bounding box can be rectangular, circular or even irregular. IoU essentially measures the degree of overlap between the predicted box and the ground truth (see **Figure 5B**). It is calculated as the area of the intersection/overlap divided by the area of the union. IoU ranges between 0 and 1 with 0 for no overlap and 1 for perfect overlap. For a given threshold α, the detection is said to have a true positive if IoU>α, and a false positive if IoU≤α. A false negative detection is when the ground truth is not detected.

In addition to the importance of the context and interdependencies of the above performance measures, the reliability of each of these rendered metrics is also an essential step for any ML model assessment task. In general, the reliability of the performance measures is directly tied to the sample size (i.e. larger sample size are more reliable than smaller ones). There are a variety of ways to assess the reliability of the above performance measures but the most common ones within ML are the confidence interval and the p-value (described below).

## 3 Reliability assessment of the performance metrics

While it is intuitive to rank ML models based on a given performance metric, one should also consider the statistical reliability and uncertainty in estimating the metric. Confidence intervals (CI) are commonly used to quantify such reliability in ML studies. For instance, a 95% confidence interval imply that the true metric will fall into this interval with a 95% probability. The computation of the 95% confidence intervals is generally expressed as


[Metric estimate−1.96×SE, Metric estimate+1.96×SE]

when the distribution of the metric follows or approximates a Gaussian distribution. SE represents the standard error of the metric estimate, and 1.96 is the quantile from the standard Gaussian distribution. Other quantiles may be used for different confidence intervals. The form of SE depends on the metric being used. For example, the SE of the R^2^ statistic for regression can be computed as $SE_{R2}=\sqrt{\frac{4R^{2}{\left(1-R^{2}\right)}^{2}\left(N-p-1\right)}{\left(N^{2}-1\right)\left(N+3\right)}}$ (19). For accuracy, the SE can be computed as $SE_{acc}=\sqrt{\frac{Accuracy\left(1-Accuracy\right)}{N}}$ , and this SE formula also applies to other classification metrics expressed as proportions (e.g., sensitivity and specificity). In some cases, the sampling distribution of the evaluation metric is no longer Gaussian (e.g., a small sample size), for which the bootstrap resampling method can be used to compute the confidence intervals (20). A larger sample size usually yields a smaller SE and thus a narrower confidence interval, implying a more precise estimate.

In hypothetical scenario 1 with a sample size of 100 (**Supplementary Figure 3**), model 1a has an accuracy of 82% (95% CI, 73.05% - 88.97%) and the accuracy for model 2a is 86% (95% CI, 77.63% - 92.13%). Although model 2a has a higher accuracy, its confidence interval contains 82%, the accuracy of model 1a. Therefore, model 2a’s improved performance over model 1a is not statistically significant. On the contrary, in scenario 2 with a much larger sample size of 10000, the 95% CI for the accuracy of model 2b shrinks to [85.3%, 86.67%], where its lower bound is greater than the upper bound of the CI for model 1b (82.75%). In this case, it can be said that model 2b’s improved performance over model 1b is statistically significant.

The formal comparison of a performance metric between two models can also be formulated as a statistical hypothesis testing problem, where the null hypothesis states that the two models have same performance, and the alternative hypothesis indicates that two models have different performance (two-sided test) or one is better than the other (one-sided test). A *p*-value less than the prespecified type I error, usually 0.05, rejects the null hypothesis and the alternative hypothesis is then concluded with statistical significance. A p-value greater than 0.05 indicates insufficient evidence to reject the null hypothesis. However, since the two competing models are trained on the same dataset, their performance metrics are correlated with an unknown correlation structure. Therefore, there are usually no dependable closed forms to compute the p-value, and the standard paired t-test or chi-squared test is usually not the best approach. However, in some cases when needed, the p-value can be derived from the 95% CI with certain limitations (21). There are also some recent developments based on cross validation or Bootstrap to derive p-values for comparing performance metrics of different ML models (22–24).

## 4 Practical considerations

### 4.1 Data preprocessing

Real-world data for ML are often noisy and contain missingness and much redundant information. Without appropriate data preprocessing, it could be very difficult to successfully train an optimized and generalizable ML model. Some common preprocessing steps include data cleaning, normalization, transformation, and dimension reduction. Data cleaning typically addresses inconsistencies, outliers, missing values, etc. Highly skewed features sometimes also need to be transformed or discretized. Normalization is performed to scale the numerical features so that their values are at the same magnitude, which is a prerequisite for many ML models such as the nearest neighbor approach and neural networks. Dimension reduction involves removing multicollinearity and redundant features, which may help reduce the noise that could have deteriorated the model’s performance. Data preprocessing is very important because it can have a significant impact on the statistical performance of a model. Ultimately, the appropriate data preprocessing steps can help improve the generalizability of the ML model of interest.

### 4.2 Model generalizability and cross validation

One of the major challenges of supervised learning is overfitting, in which case a model yields very satisfactory or even perfect performance on the trained data but performs poorly when applied to yet-unseen data. In other words, the model has low generalizability. Ideally, to obtain an accurate understanding of the model’s performance, the model should be evaluated on an independent dataset, which is referred to as a test or generalization set in the literature. However, in many cases, researchers don’t have immediate access to such additional data. A heuristic solution is to split the available single dataset into two separate datasets (one used for the training and initial validation and the other used for the generalization test). Typical recommendations for the split ratio are 60%-40%, 70%-30% or 80%-20%, depending on a multitude of factors include but are not limited to the number of classes and target class sizes. The split can be completely random or be stratified by the output to maintain same distribution of the outcome in the training and initial validation test sets. The ML model is then estimated from the training set (e.g. random 70% in the 70%-30% split noted above). Once training is completed, the model is applied to the features in the validation test dataset (e.g. remaining 30% in the 70%-30% split noted above). The predictions made by the algorithm are then compared to the known outcomes of the validation test dataset to assess the model performance. Such an approach is referred to as internal validation. While this approach is conceptually simple and easy to implement, the sample sizes for both the training and initial validation test sets can be significantly reduced after split, especially for small datasets, and then the results may depend on a particular split of the dataset. To further enhance the reliability of the performance metrics, cross validation can be integrated within this “train-test split” process.

The classic *k*-fold cross validation (CV) randomly divides the dataset into *k* groups of approximately equal size. The model is then trained on *k*-1 folds of the data, and the left-out fold is used to evaluate the model performance. This procedure is repeated *k* times so that each fold is treated as a validation set once. The *k*-fold CV results are then averaged across the *k* test sets. For example a *k*-fold CV of 10 will have trained 10 separate “train-test split” models whose initial validation performances are then averaged to get a better sense of the model’s true capabilities. Some research has shown that *k*=10 yields similar performance to leave-one-out cross validation which is the most extreme approach (i.e., *k*=sample size minus 1) (25). In summary, cross validation provides an estimate of the performance of a model on unseen data and it is a fundamental step in the model selection and hyperparameter tuning process within machine learning. It also allows to identify if a model is overfitting or underfitting by comparing the performance on the training and validation test sets. Such cross validation allows for a more robust estimation of model’s true capability by averaging its statistical performance on several different train-test split versions of the data (as noted above which results in a better representation of the final model’s true performance, i.e. more likely generalizable and less chance of being overfitted).

Although this helps the generalization of the ML model, but for small to intermediate datasets (which is a great deal of datasets within medical studies), this process by itself is insufficient to declare a model generalizable. For such cases (small to intermediate datasets), additional testing (i.e. secondary and tertiary generalization test sets) is a necessity to render such models as potentially generalizable.

### 4.3 Model selection

Due to the rapid growth of computing power, investigators nowadays are able to build multiple supervised ML models for a given dataset efficiently. Modern automated ML (AutoML) platforms such as Auto-Keras (26) and MILO-ML (27) can generate thousands of ML models based on various combinations of algorithms, tuning parameters, and pipelines without human intervention. However, selecting a final best model remains a complex question to address. While there are multiple evaluation metrics available as described in Section 2, they measure the ML model performance from different perspectives and thus there is not a single metric translatable to all studies. The choice of the evaluation metric should depend on the specific aims of each study, and sometimes it may rely on multiple metrics. In practice, in addition to the performance metrics, there could be other competing factors such as the complexity and cost to deploy and maintain the model. Thus, a comprehensive examination is required for any model selection.

### 4.4 Sample size determination for supervised ML

No consensus has been established about how to determine the best sample size for supervised learning (28–30). Various “rules of thumb” have been proposed and debated. However, for regression-based supervised models, some recent studies developed guidelines and statistical formulas for calculating the sample sizes based on specific prediction metrics through internal validation (28, 29, 31). Simulation can be also used to estimate prediction performance by varying the sample sizes, and a final sample size is determined as the number achieving a desired performance (e.g., 80% accuracy). Another generic approach for binary classification is to fit an inverse power law model to points of a given learning curve created using the available data (32). The fitted model is then used to predict the classifier’s performance for larger sample sizes. Additionally, the data sample size can drastically change the statistical reliability of the ML model’s performance measures (e.g. accuracy of a model based on 1 million cases will have a much tighter 95% CI than one that was based on 1000 cases). Therefore, for more limited data studies, additional test sets (i.e. secondary and tertiary testing) is a must since without which the chance of developing an overfitted (not generalizable) ML model is drastically increased (33).

## 5 Discussion

ML has become a significant integrated component of healthcare in recent years. Many supervised ML models have been developed for early detection of cancer, disease diagnosis and prediction of patient outcomes. These ML algorithms are able to read all kinds of features in healthcare, including patient demographics, clinical information, laboratory tests, genetic variants, texts, and medical images (e.g. histology and radiology images). Advanced ML models can further integrate features from these different domains for multi-modality analysis. ML models are also being transferred into wearable devices and smartphones, which enables patient care activities outside of the hospitals such as in outpatient or at-home settings (3).

However, the development of ML tools in healthcare is no trivial task and faces many challenges. One major challenge is the access to high quality data. Most healthcare data are acquired from patients, which are governed by stringent regulations such the Health Information Portability and Accountability Act (HIPAA) in the United States and the General Data Protection Regulation (GDPR) in Europe. Even when such data are available, investigators need to submit appropriate proposals to regulatory committees (e.g., institutional review board) to ensure adequate protection of the data and patient privacy before conducting research. A recent report from the US Government Accountability Office identifies data availability as a main barrier to the application of AI or ML in healthcare (34). The issue of data availability also has significant impact on the generalization of ML models. Most studies use a single dataset for model development and validation. Although as discussed earlier, internal cross-validation may reduce the risk of overfitting to some extent, an external validation (i.e. secondary and tertiary generalization testing) is essential in most cases to examine the true generalization performance of the ML model. A related issue is data heterogeneity in the sense that the same set of variables from different datasets or even different institutions could be measured differently, leading to unsuccessful reproducibility of the ML models in practice (35). Furthermore, data quality issues such as missingness or measurement error often require a large amount of effort to preprocess the data. Another challenge is the computational cost, especially for studies with large sample sizes, where significance computing resources (e.g., memory, graphical and computer processing units, and storage) are demanded. One solution could be the use of synthetic data to expedite and enhance the various challenges described above. Our advancements in computational sciences are now enabling us to create synthetic datasets that can mimic the performance of their real data counterparts.

ML and AI have the great potentials to improve and transform healthcare in the near future. ML will not only improve patient care outcomes, but can also help significantly reduce the healthcare cost and improve healthcare system operational activities. By understanding the ML concepts, algorithms, and the related statistical performance metrics, along with the opportunities and challenges, all healthcare professionals and researchers will be able to play pivotal roles within this coming transformation.

## Author contributions

HR and BH: concept and design; HR, SA, SR, NT, and BH: analyze and interpret the data; HR and BH: writing and finalizing the paper; SA, NT, and SR: commenting and revising the paper. All authors contributed to the article and approved the submitted version.
