# Supplementary figures and images for: Common statistical concepts in the supervised Machine Learning arena
Source: Front Oncol. 2023 Feb 14;13:1130229. doi: 10.3389/fonc.2023.1130229 (PMC9949554; doi:10.3389/fonc.2023.1130229)

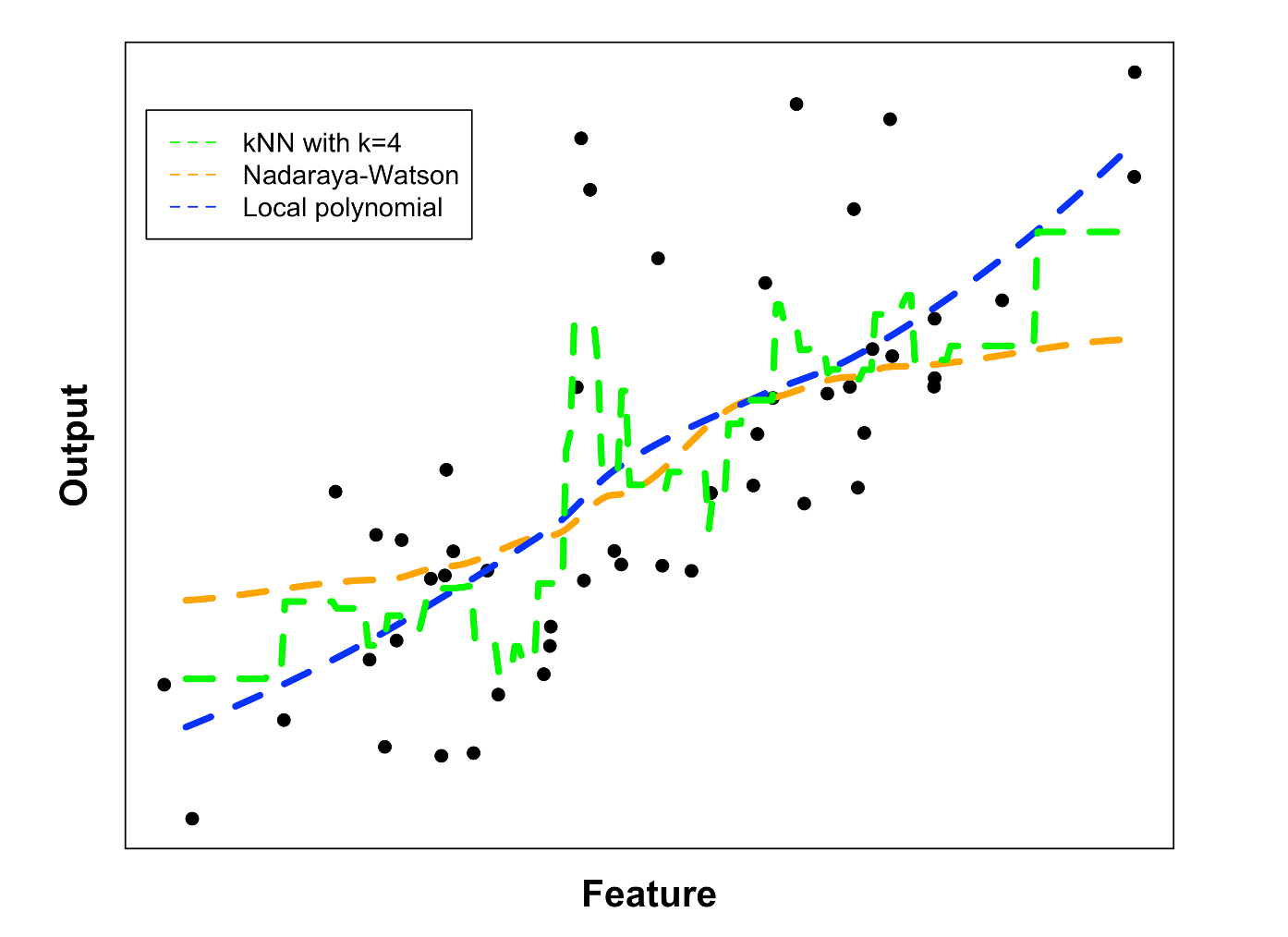

Supplement: Supplementary file 1 [file Image_1.tiff]

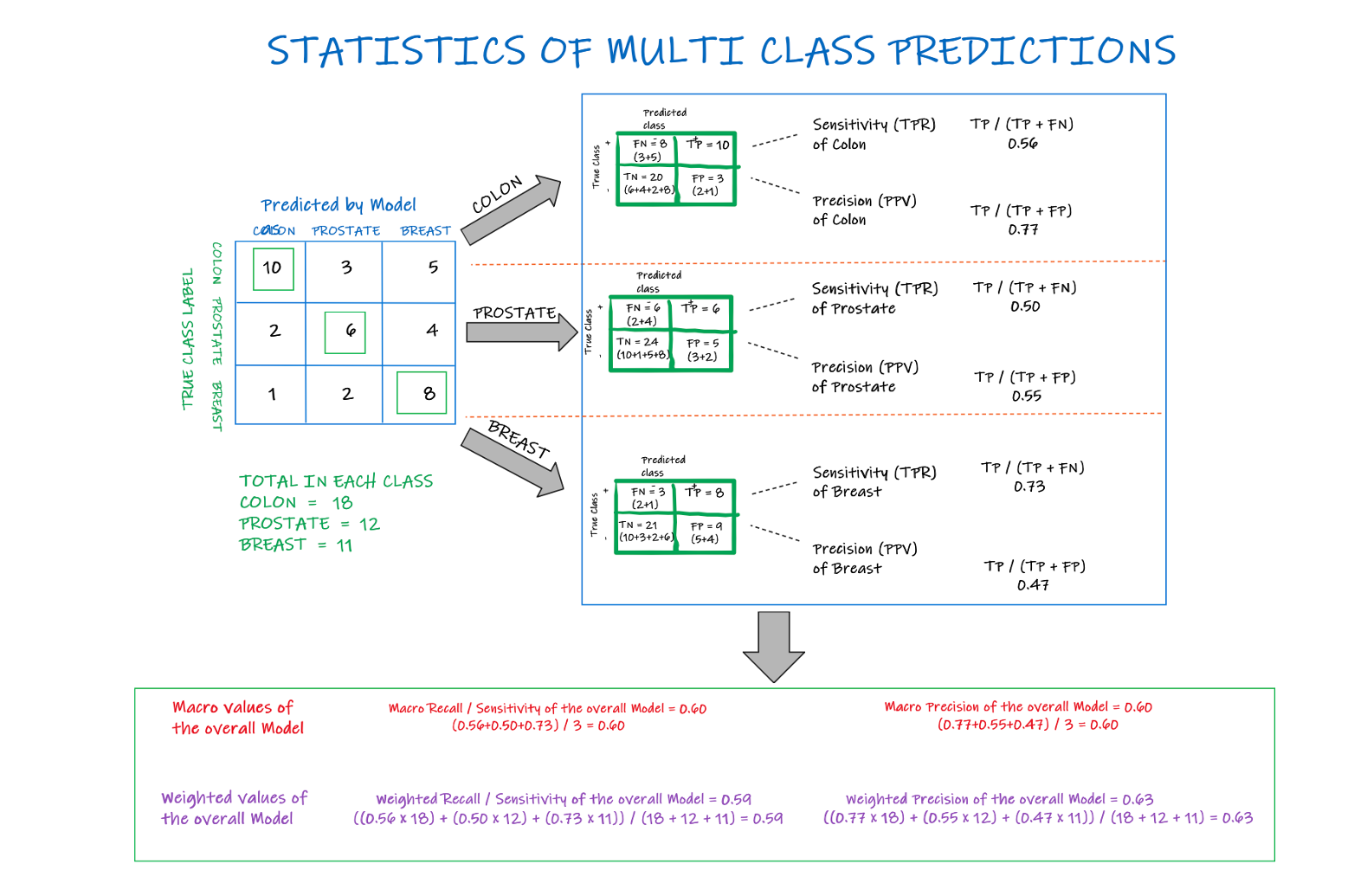

Supplement: Supplementary file 2 [file Image_2.tiff]

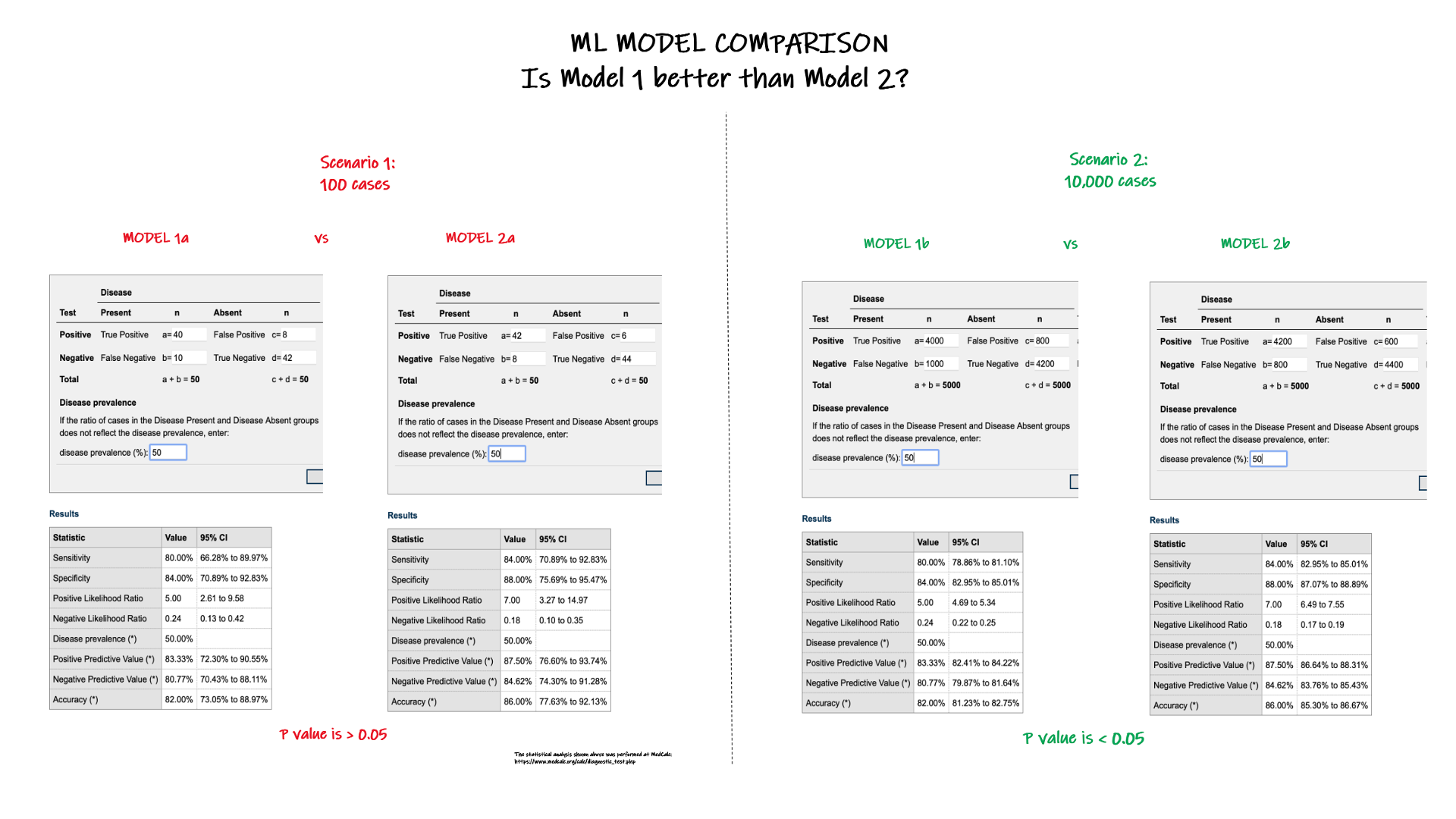

Supplement: Supplementary file 3 [file Image_3.tiff]
