# Supplementary material for: Common statistical concepts in the supervised Machine Learning arena
Source: Front Oncol. 2023 Feb 14;13:1130229. doi: 10.3389/fonc.2023.1130229 (PMC9949554; doi:10.3389/fonc.2023.1130229)
Supplement: Supplementary file 4 [file Table_1.docx]

Supplementary Table 1. Matthews correlation coefficient (MCC) and Cohen’s kappa for multiclass classification

| Metric | Formula |
| --- | --- |
| MCC | $\frac{n_{c}\times N-\sum_{k=1}^{K} c_{k}r_{k}}{\sqrt{(N^{2}-\sum_{k=1}^{K} c_{k}^{2})(N^{2}-\sum_{k=1}^{K} r_{k}^{2})}}$ |
| Kappa | $\frac{n_{c}\times N-\sum_{k=1}^{K} c_{k}r_{k}}{N^{2}-\sum_{k=1}^{K} c_{k}r_{k}}$ |

$n_{c}$ is the total number of correct predictions (sum of the diagonal elements); $N$ is the total sample size; $c_{k}$ is the total number of samples with predicted class *k* (column total); and $r_{k}$ is the total number of samples in class *k* (row total).
